# Consumer Preferences for Animal Welfare in China: Optimization of Pork Production-Marketing Chains

**DOI:** 10.3390/ani12213051

**Published:** 2022-11-06

**Authors:** Yaoming Liang, Yu Cheng, Yanjie Xu, Gengrong Hua, Zijian Zheng, Hui Li, Li Han

**Affiliations:** 1 College of Economics & Management, South China Agricultural University, No. 483 Wushan Road, Tianhe District, Guangzhou 510642, China; 2 College of Veterinary Medicine, South China Agricultural University, Guangzhou 510642, China; 3 School of Sociology and Anthropology, Sun Yat-sen University, 135 Xingang Road, Haizhu District, Guangzhou 510275, China; 4 Faculty of Social Sciences, University of Macau, Avenida da Universidade, Taipa, Macau 999078, China; 5 College of Public Management, South China Agricultural University, Guangzhou 510642, China; 6 College of Natural Resources and Environment, South China Agricultural University, Guangzhou 510642, China

**Keywords:** animal welfare, consumer preferences, willingness to pay, choice experiment

## Abstract

**Simple Summary:**

In this study, consumer preferences for pork produced using animal-welfare-enhancing farming strategies were assessed. In China, the demand for animal-friendly products is increasing, but so far, studies on consumer preferences for animal welfare farming attributes are limited. The objective of this study was to analyze consumer preferences for pork based on four animal welfare farming attributes, namely feed nutrition, living environment, health care, and activity space. The study employed a choice experiment approach. The survey covered 1274 pork consumers in Guangdong province, China. Our empirical results suggest that Chinese consumers were willing to pay an additional 2.359–10.477 CNY/500 g (5.27–23.39%) for animal welfare pork. Furthermore, there was significant heterogeneity in consumer preferences. China is the world’s largest producer and consumer of pork. Since the outbreaks of African swine fever in 2018, China’s pork imports have been constantly on the rise. The results can contribute to the optimization of pork production structures and marketing plans for stakeholders and can assist with the timely development of international competition strategies for animal-derived trade products.

**Abstract:**

Consumption demands for pork produced by farms that employ strategies to improve animal welfare (“animal welfare pork”) will be an important indicator for predicting domestic pig feeding standards and pork industry development. This paper analyzes consumer preferences for animal welfare pork based on the choice experiment data of 1274 pork consumers in Guangdong province, China. The results show that consumers had a significant preference for animal welfare pork and that they were willing to pay a premium of 2.359–10.477 CNY/500 g (5.27–23.39%) on average. There is heterogeneity in consumer preferences regarding age, education level, and income. Producers of animal-derived products can not only adjust the mix of production conditions to improve pig welfare and innovate contractual arrangements for industry chain stakeholder groups, but they can also develop differentiated marketing strategies for animal welfare products to meet consumer demands for animal welfare.

## 1. Introduction

There is a large international debate regarding the welfare of animals raised for food [1]. Many scholars consider animal welfare to be a positive attribute of food [2]. According to the Terrestrial Code of World Organisation For Animal Health (WOAH), animal welfare refers to: ”the physical and mental state of an animal in relation to the conditions in which it lives and dies’’. It is a branch of science and looks at these measurable states in almost all areas of our interaction with animals, including areas of agriculture, entertainment, companionship, research, and others [3]. Studies have shown that animal welfare can not only have a positive impact on the growth and health of farm animals [4,5], thereby improving the quality of animal-derived products, but that it also helps meet ethical and moral requirements of the public. If the living environment can be modified according to the physiological and behavioral habits of different animals, consideration of the concept of animal welfare will undoubtedly reduce animals’ stress; improve their immunity, fitness, and health; and reduce the use of pesticides, feed additives, and veterinary drugs that pose serious health hazards to consumers. Improving animal welfare may become an important issue in the breeding industry.

**Figure 1:**
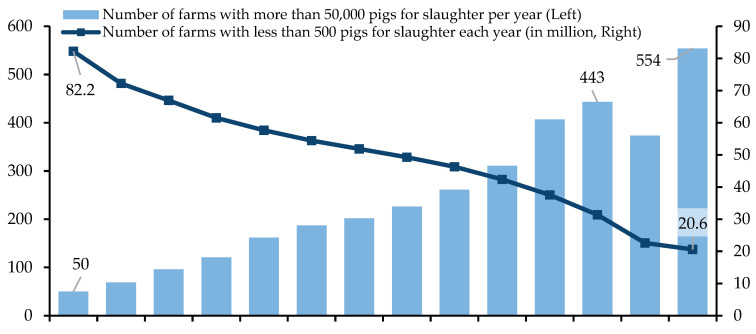
Number of Pig Farms in China from 2007 to 2020. (Source: China Animal Husbandry and Veterinary Yearbook).

China is a major producer and consumer of pork worldwide, and pig rearing is the backbone of the domestic livestock industry (pork production in China has reached 52.96 million tons in 2020, accounting for more than 59.6% of meat production according to the 2021 China Statistical Yearbook). With the optimization of industrial structures, farms that produce fewer than 500 pigs per year decreased from 82.2 million in 2007 to 20.6 million in 2020, while farms that produce more than 50,000 pigs per year increased from 50 in 2007 to 554 in 2020 (Figure 1). The issue of animal-derived food safety and farm animal welfare caused by intensive pig farming has become an urgent concern. In May 2014, China introduced the Farm Animal Welfare Requirements for Pigs, the first farm animal welfare standard concerning advanced foreign farm animal welfare concepts concerning the existing domestic scientific, technological, and socio-economic conditions. According to the requirements, the whole process of animal welfare management regarding pigs’ breeding, transport, slaughter, and processing is to be regulated as it relates to aspects such as feed and drinking water, farming environment, farm management, health plans, transportation, slaughter, splitting and processing, records and traceability, etc. This increases the possibility of exploring issues related to pig welfare in China.

The future of agriculture depends in large part on consumer demand. It is critical for public health departments and animal-derived product marketers to understand consumer preferences and willingness to pay (WTP) for animal welfare. According to the results of a survey conducted by You et al. (2014) among 6006 consumers in 29 provincial administrative regions in China (excluding Tibet, Hainan, Taiwan, Hong Kong, and Macau), 54.5% of the respondents were willing to pay a higher price for animal welfare pork at least to some extent [6]. Wang and Gu (2014) found that consumers in Jiangsu province were willing to pay an average of over 16.2% of the base price for animal-friendly pork before being given information about the association between animal welfare and meat quality, and they were willing to pay over 21.3% of the base price after being given information about the association [7]. At present, most of the studies on Chinese consumer preferences for farm animal welfare still remain at the level of descriptive statistical analysis. Only a few studies, such as Wu et al. (2014) [8] and Xu et al. (2019) [9], focus on empirical methods. Additionally, studies on consumer preferences for animal welfare breeding attributes are uncommon in China.

This paper focuses on how much of a premium consumers are willing to pay for animal welfare pork in China. The study mainly covers the following aspects. First, the paper incorporates a limited choice experiment examining consumer preferences for animal welfare pork based on four attributes: feed nutrition, living environment, health care, and activity space. Second, it helps the stakeholders better understand the heterogeneity in Chinese consumer preferences for animal welfare farming methods, and it provides theoretical support for the formulation of farm-animal-management-related policies and marketing strategies for livestock products in China. This not only helps domestic stakeholders grasp the dynamic consumer preferences and optimize production structures and marketing plans, but it also improves the world’s understanding of China’s huge animal-derived food consumption market and the development of international competition strategies for agricultural trade products.

The structure of this paper is as follows. The choice experiment and survey design, estimation methods, and sample data are explained in Section 2. Empirical results are analyzed in Section 3. Discussions and policy implications are considered in Section 4. Finally, conclusions are presented in Section 5.

## 2. Research Methodology

### 2.1. Choice Experiment Design

In China, consumers do not know much about animal welfare, and there are no products with animal welfare labels on the market. Consumers tend to choose animal foods to meet their consumption needs by considering the manner, conditions, and environment in which animals are raised (e.g., whether they have a healthy diet, a good living environment, scientific health care, and adequate space to move around). Due to the lack of a real market, consumer-preference-assessment methods based on actual market prices are not suitable for evaluating consumer demand for non-market product attributes. However, hypothetical choice experiments can be advantageous in such cases and are thus widely used in this regard [10]. In particular, choice experiments have been widely used to measure consumer preferences for food with certain attributes, e.g., Wu et al. (2016), Ortega et al. (2017), Wang et al. (2018). Kallas et al. (2019), Czine et al. (2020), Huang et al. (2022), Lin-Schilstra et al. (2022) [11,12,13,14,15,16,17].

Pig welfare products can be seen as a collection of different welfare attributes from which consumers can obtain utility. Choice experiments enable evaluation of multiple attributes by replicating real life shopping scenarios [18]. Using this approach to determine consumers’ willingness to pay has become a better way of assessing consumer demand and animal welfare preferences [19]. It provides important information to policymakers or marketers who are preparing and implementing such certification systems and promoting them widely [20]. Such information would be useful for stakeholders in the supply chain for designing production processes and developing marketing strategies based on these production attributes, and it would be valuable for developing appropriate marketing communication tools [21].

In order to meet social concerns about the welfare quality of animal food and the related market demand, and to promote access to products that meet specific animal welfare standards, the EU published a welfare quality assessment scheme in 2009 for three categories of farm animals: pigs, cattle, and poultry. The standards of animal welfare practices are assessed in four areas: good feeding, good housing, good health, and appropriate behavior. Accordingly, we designed the choice experiment of animal welfare consumption preference with reference to the above four aspects. Specifically, we are more concerned about such aspects as feed nutrition, living environment, health care, and activity space, which seem to be the more prominent problems in China’s pig industry [22,23,24].

It is worth noting that providing activity space and outdoor access are the two most important attributes necessary to obtain an acceptable level of welfare, as this prevents injuries and suffering [25]. Most consumers share this concern and cite the permission of outdoor access as a very important characteristic of pig welfare [26]. In terms of activity space, this paper refers to the welfare levels set up by Denver et al. (2017) [27], in which outdoor access is considered in addition to increased activity space. Therefore, the activity space attribute is defined in three levels, while the other three attributes are defined in two levels.

The price consists of four levels. The reference is based on the average market price of lean pork loin in large, medium, and small supermarkets as well as wet markets and online fresh food platforms (JD Fresh, Suning Commerce and Fresh Hema) in Guangzhou city (the capital city of Guangdong province) in February 2020. The pricing strategy of product marketing was also considered (e.g., any price ending with the number “8” is considered as a lucky number to Chinese consumers, as “8” has a similar pronunciation with “fa”, the Chinese character of “wealth”). The animal welfare attributes and their levels in the choice experiment are shown in Table 1.

**Figure 2:**
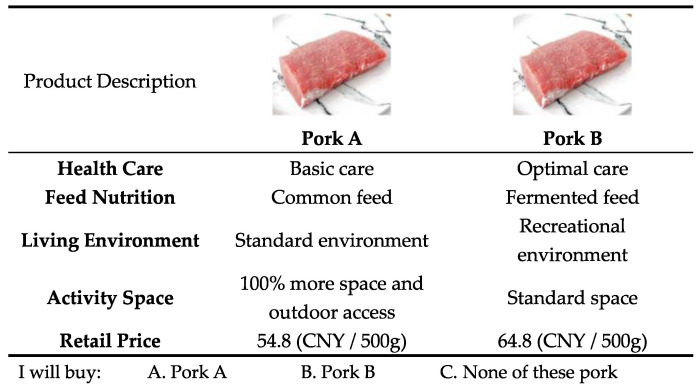
Sample of a choice set.

In this paper, 24 choice sets were designed to estimate consumers’ utility of animal welfare pork based on a D-optimal fractional causal analysis experimental design using the Ngene 1.2.1 software package (www.choice-metrics.com, (accessed on 16 September 2022)). The 24 choice sets were divided into four groups and each respondent was randomized to complete one of the groups for a total of 6 choice sets. In this way, respondents may have the ability to complete the entire choice experiment within a reasonable time frame. It is generally accepted that providing an “opt-out” or “no purchase” option in the choice set more closely resembles the real decision scenario [28]. Therefore, each choice set includes the following three options with different animal welfare farming attributes: Pork A, Pork B, and neither. Figure 2. illustrates one of these choice sets.

### 2.2. Survey Design

Targeting pork buyers is a key part of the survey because targeting ordinary consumers may lead to bias in estimation caused by sample selection. Guangdong province was selected as the survey area for the following reasons. First, Guangdong’s economic and social development level is among the highest in the country, with the GDP reaching about 1.92 trillion USD (Exchange rate: 1 USD/CNY 6.1798, December 31, 2021 (CFETS).) in 2021 and ranking first for 33 consecutive years in China. Second, Guangdong, adjacent to Hong Kong and Macau, is a major province of foreign trade, where people may be more likely to accept the concept of animal welfare. Third, there is a common belief that “people in Guangdong know about food” in China. Furthermore, consumers aged 16 and above were targeted as respondents in the survey, as China implements a nine-year compulsory education system, and 16 years old is usually the corresponding age for an individual to complete their compulsory education. Some previous research has included the 16-year-old group in survey subjects when assessing the purchasing of household food items, including Liu & Niyongira (2017), Liang et al. (2023) [29,30].

In developing countries, new ideas such as concern for animal welfare are generally easily accepted among young people with higher levels of education and better economic incomes [31,32]. In today’s new media era, this group has access to a large amount of information via smartphones. Since a face-to-face survey method would substantially increase the cost of the survey and could lead to bias caused by limited consumer cognitive resources (time and energy) at the time of the survey, and because of the impact of the global COVID-19 pandemic, the data for this paper were obtained through an online survey.

The definitions of farm animal welfare and its products were clearly given in the guidelines of the questionnaire. The choice experiment was conducted immediately after respondents answered some basic questions about their perception of farm animal welfare and its products. In the survey, they were presented with a “cheap talk script” about the choice experiment designed to reduce their hypothetical bias [33,34]. The term “cheap talk” is borrowed from experimental economics, where it refers to communication between players prior to execution of an experiment. Here, a cheap talk script refers to open communication between the experimenter and the respondents about things to consider when responding to a subsequent question. The cheap talk script was followed by a description of the information about animal welfare farming attributes.

To ensure randomization of the survey, respondents were assigned to different groups of purchase scenarios based on the parity of the last two digits of their cell phone number (two odd numbers, two even numbers, odd followed by even, even followed by odd). They were only able to see one choice scenario at a time in order to exclude interference from other choice scenarios. They spent at least 15 s in each choice scenario to ensure an acceptable quality of data. In addition, the order of the six choice scenarios faced by each respondent was randomized to exclude any order effects on the estimated results. The choice experiment was followed by a survey on respondents’ pork consumption habits and basic personal information. The questionnaire would be completed in about 15 min.

To reach statistical significance and satisfy the rank condition of the choice experiment, we adopted a protocol generally used in choice experiments design [35,36,37] to determine the minimum sample size:

$$N\geq 500\times \left(\frac{L}{A\times C}\right)=500\times \left(\frac{4}{3\times 6}\right)=111.111$$

Here, N is the number of the sample; L is the largest number of levels of any of the attributes; A is the number of choice options in a choice set; and C is the number of choice sets faced by each respondent. Given that we divided the 24 choice sets into four groups, the minimum sample size for this choice experiment would be 112.

### 2.3. Estimation Methods and Econometric Models

This paper uses a random utility model to analyze consumer preferences. The choice experiment is based on the following assumption: individual *n* obtains utility by choosing option i from a finite set of alternative options J of choice set C under scenario t. In the random utility model, utility consists of a deterministic component $V_{nit}$ that depends on the attributes of the options and a random component $\varepsilon_{nit}$, i.e.,

(1) $$U=V_{nit}+\varepsilon_{nit}$$

Thus, if $U_{nit}>U_{njt}\;\forall j\neq i$, then individual n will choose option i. Consequently, the probability that individual n will choose option i is

(2) $$P_{nit}=Prob(V_{nit}+\varepsilon_{nit}>V_{njt}+\varepsilon_{njt}\;;\;\forall j\in C,\;\forall j\neq i).$$

Given the underlying distribution of the error term, the final form of the logit selection probability can be expressed as:

(3) $$P_{nit}=\frac{\exp\left(V_{nit}\right)}{\sum_{j}\exp\left(V_{njt}\right)}$$

While traditional logit models assume that consumers are homogeneous, the random parameter logit (RPL) model relaxes the constraints of traditional logit models by allowing random variation in in-sample preferences according to a specified distribution [38]. Accordingly, the RPL model can be used to measure heterogeneity in consumer preferences for animal welfare farming attributes. Based on the RPL model, the deterministic component of utility $V_{njt}$ in the random utility model takes the following form:

(4) $$V_{njt}=\beta^{\prime }\chi_{nit}$$

where β is a vector of random parameters with their own mean and variance indicating individual preferences and $\chi_{nit}$ is a vector of all attributes in the *i*th choice. According to Train (2003) [39], the probability that individual n will choose option i from the choice set *C* under scenario t is:

(5) $$P_{nit}=\int^{}\frac{\exp\left(V_{nit}\right)}{\sum_{j}\exp\left(V_{njt}\right)}f\left(\beta \right)d\beta$$

where the random parameter f( ) of the distribution is specified. If the parameter is fixed to $\beta_{c}$ (non-random), the distribution fails, i.e., $f\left(\beta_{c}\right)\to \infty$, otherwise f(β)=0.

Considering that utility is non-basic in nature and that the estimated model coefficients cannot be interpreted in economic terms, the willingness to pay of consumers is estimated as:

(6) $$WTP=\frac{-\beta_{k}}{\beta_{p}}$$

where $\beta_{k}$ is the estimated coefficient of the *k*th attribute and $\beta_{p}$ is the estimated price coefficient. A 95% confidence interval was created using a parametric bootstrap procedure as suggested by Krinsky and Robb (1986) [40]. Specifically, a multivariate normal distribution was created by parameterizing the coefficients and variance terms estimated using the RPL model, from which 1000 observations were extracted.

### 2.4. Sample Source and Data Description

This survey was anonymous and ethical approval was granted by College of Veterinary Medicine, South China Agricultural University. A pre-survey was conducted in February 2020. We rephrased the questionnaire to make it more concise and easier to understand, removed survey questions inconsistent with the local situation, and added some more valuable questions based on the feedback and suggestions from 90 sample consumers.

Thereafter, a formal investigation was conducted in March 2020 via the paid online platform provider Wenjuanxing, which is a professional online survey platform in China that focuses on providing users with services such as powerful, user-friendly online questionnaire design, data collection, custom reports, and survey result analysis. The platform recruits and maintains a group of consumers who participate in surveys from time to time with small incentives. Participants will randomly receive email invitations and URLs directing them to the survey, and they subsequently receive rewards in the form of credits that can be converted to vouchers for shopping. Participation in each investigation is voluntary. The sample service of Wenjuanxing provides strict quality-control mechanisms, including sample quality control, filler control, filling process control, the whole tracking effect, etc., to ensure recovery of true and valid response data.

Finally, a total of 1637 questionnaires were collected, and 1274 respondents completed the entire survey. This produced a sample of 7644 choices (1274 respondents × 6 choice sets). The choice experiments included a significant number of pork consumers, which allowed us to investigate consumption preference and heterogeneity. All statistical analyses were carried out using the software package Stata 16.0 (Stata Corp. 2019, Stata Statistical Software: Release 16, StataCorp LLC, College Station, TX, USA).

The demographic characteristics of the sample consumers in Table 2 show that the proportion of females (62.48%) is significantly higher than that of males (37.52%), which is consistent with the fact that more women are responsible for taking care of the family’s food. The average age of the respondents is 32.2 years old, and more than 50% have a university degree or higher, indicating that the sampled consumers are younger and more highly educated. Nearly 70% of the surveyed households have a monthly income between 6000 and 24,000 CNY. Nearly 50% of the households have children eating with them. Nearly 40% of the households have elderly people eating with them.

## 3. Results

### 3.1. Consumer Preferences for Welfare Attributes of Fattening Pigs

As shown in Table 3, there is a significant preference for animal welfare pork. Consumers have the highest preference for the attribute of providing 100% more space and outdoor access. They are willing to pay a premium of 10.477 CNY/500 g (23.39%) compared to that of providing indoor rearing space in accordance with the national standard. However, their willingness to pay for 100% more rearing space is relatively low (a premium of 2.359 CNY/500 g or 5.27%), which suggests that Chinese consumers prefer the farming method of “free range” over simply increasing indoor rearing space. The premiums for the attributes of optimal care and fermented feed are 6.689 and 5.893 CNY/500 g, respectively. The premium is the lowest (2.560 CNY/500 g) for the attribute of providing recreational facilities such as toys and music instead of merely providing a ventilated, clean, and odor-free living environment.

Table 3 also shows that the standard deviation coefficients of all three variables (fermented feed, optimal care, and recreational environment) are significant at the 1% level except the “activity space” variable. It also indicates the heterogeneity of consumer preferences for pig welfare farming attributes. Specifically, consumer preferences for the recreational environment attribute are the most varied, with a standard deviation coefficient of 0.754, followed by the optimal care attribute (0.434) and the fermented feed attribute (0.372). This suggests that heterogeneity should be considered in studying consumers’ preferences for animal welfare farming attributes, and the hypothesis of using the RPL model to analyze consumer preferences for animal welfare is confirmed.

### 3.2. Heterogeneity Analysis of Consumer Preferences

As mentioned previously, there is heterogeneity in consumer preferences for animal welfare pork. It is practically relevant for pork producers and marketers to visualize consumer preferences for farm animal welfare through demographic characteristics. The random utility model shows the difference in utility brought to consumers by different product options rather than the absolute value of utility brought by a single product. Hence, the effect of individual consumer characteristics on utility is usually omitted directly in the expression of the function because individual characteristics do not vary with product options [39]. A common approach is to set interaction terms between consumer socio-demographic characteristics and product attribute levels in the model to analyze the effect of consumer characteristics on consumer preferences or willingness to pay, as done in Wu et al. (2014) and Wu et al. (2016). Following this approach, we formed interaction terms to examine how demographic variables affect consumption preferences related to animal welfare.

According to the age distribution, the sample can be divided mainly into three groups: ≤25 years, 26–35 years, and ≥36 years. The proportions in these groups are 33.12%, 36.74%, 30.14%, respectively. Some of these age nodes can be found in the existing literature, such as Lim et al. (2013), Wu et al. (2015), Han et al. (2015), and Denver et al. (2017) [8,27,41,42]. Education levels of primary school and below; junior high school; high school/technical secondary school; college/higher vocational; and undergraduate, postgraduate, and above correspond to 6, 3, 3, 3, 4, and 3 years of education, respectively. Furthermore, we divided the sample into high-income and low-income categories based on income distribution, which accounted for 50.31% and 49.69% of respondents, respectively. In summary, the above socio-demographic variables can be classified as follows:
Gender:female, male;Age:low age (≤25), middle age (26–35), and advanced age (≥36);Education:high education (≥16 years) and low education (<16 years);Income:high income (≥12,000 CNY) and low income (<12,000 CNY).

Accordingly, they formed interaction terms with each attribute variable of pig welfare, and RPL model regression estimation was conducted separately. Strictly speaking, the experimental design has to be adjusted after the introduction of the interaction effect, and the design scheme that only considers the main effect will lead to inefficient estimation [21]. In this paper, the interaction effects between attributes were not considered in the experimental design. In addition, we considered only one interaction term of consumer characteristics when conducting the RPL model estimation in order to avoid adding too many independent variables and over-parameterization caused by crossover between all of the individual characteristic terms and attribute terms [43]. Fortunately, the significance and sign of each welfare attribute may largely be consistent with those of the baseline model in the estimated model with the introduction of the interaction term.

It can be seen from Table 4 that, except for gender, age, education level, and income, all variables significantly affect consumer preferences regarding pig welfare farming attributes, albeit to varying degrees. Compared with consumers in the low age group (25 years old and below), consumers in the advanced age group (36 years old and above) are more concerned about the expansion of activity space and outdoor access (an interaction coefficient of 0.202), while consumers in the middle age group (25–35 years old) are less concerned about optimal care for pigs. The coefficients of interaction between the variables of being highly educated and preferring fermented feed as well as between being highly educated and preferring optimal care are significantly positive (0.196, 0.126). This indicates that consumer preferences for fermented feed and optimal care can be improved with increased education. In addition, the interaction coefficient between the higher income and fermented feed variables is significantly positive (0.253), which indicates that the two variables are associated. Increasing consumer income level is linked to higher consumer preferences for the fermented feed attribute.

### 3.3. Personality Portrait Analysis of Consumers

It can be helpful for marketers to more intuitively understand the preferences of consumers for pig welfare farming attributes and to identify which groups to target for sales. In general, there are more female respondents in charge of family food shopping. It can be assumed that the consumers were female, and education level and income can be assumed to be positive correlates, i.e., higher education level would result in higher income (see Lim et al., 2013 [41]). In this paper, a total of six specific types of consumers were selected based on four socio-demographic characteristics of respondents: gender, age, education level, and income. The relative preferences of these six types of consumers with different socio-demographic attributes for each attribute of pig welfare (Table 5) were calculated and comparatively analyzed.

The results in Table 5 show that consumer preferences for animal welfare pork change with age. For female consumers in the low age group with low education and low income, their preference for pigs’ welfare is not significant. In the middle age group, those with low education and income have a significant preference for the attributes of optimal care and fermented feed (utility coefficients are 0.710 and 0.346, respectively). In the advanced age group, those with low education and income still have a significant preference for the optimal care attribute (0.359), although the degree of preference is reduced. The preference for the activity space attribute also becomes significant, especially for the outdoor access attribute, whose coefficient is the largest (0.669). It is clear that female consumers with low education, low income, and low age primarily pay attention to animal welfare attributes related to food safety and health (i.e., optimal care and fermented feed), while concern for attributes related to food quality (i.e., activity space) increases with age.

If factors such as education and income limit consumers’ actual purchases of animal welfare products, then a comparison of consumer preferences between low-education, low-income groups and high-education, high-income groups in the different age categories may help confirm this hypothesis. Table 5 also shows that the preferences of the high-education and high-income group for animal welfare pork are more or less consistent in different age groups. The utility coefficients for optimal care, fermented feed, and 100% more space and outdoor access are all significant at the 1% level, but the priorities of attributes are varied. This is somewhat consistent with the preferences of low-education, low-income people in the middle and old age groups, while the preferences of the high-educated and high-income group are stronger. Consumers in the high-education, high-income, and high age group are also concerned with the recreational environment (with the coefficient of 0.399), an animal welfare farming attribute that meets the needs of animal mental health.

It is worth noting that consumers in the low-education, low-income group, and middle age group prefer the optimal care attribute more, which seems to contradict the results in Table 4 that indicate that low-aged consumers care more about the optimal care attribute than middle-aged consumers. This may be due to the fact that the sample size of the high-education and high-income group (64.54%) is larger than that of low-education and low-income group (35.46%). Nevertheless, this illustrates the heterogeneity of consumer preferences for pork with animal welfare farming attributes. It suggests that more empirical research should be carried out regarding consumers with different characteristics.

The WTPs of the above six groups for animal welfare pork were calculated and presented in Table 5. In order to describe their characteristics more intuitively, we further assumed that the low-education and low-income group received 9 years of education and 6000 CNY per month on average while the high-education and high-income group received 16 years of education and 24,000 CNY per month.

Table 6 shows the payment premium of female consumers with different educational background, income, and age characteristics. For the low-education and low-income group, the premiums paid by middle-aged consumers for fermented feed and optimal care are 6.677 and 13.715 CNY/500 g, respectively. The premiums paid by advanced-age consumers for the attributes of 100% more space, 100% more space and outdoor access, and optimal care are 6.909, 19.335, and 10.391 CNY/500 g, respectively.

The WTPs in Table 6 also show that highly educated and high-income consumers may generally have a significant payment premium for animal welfare farming attributes. Among them, the low-age consumers are willing to pay the highest premium for the optimal care attribute, reaching 6.454 CNY/500 g. The middle-age and advanced-age consumers are willing to pay the highest premium for the 100% more space and outdoor access attribute, reaching 11.792 CNY/500 g and 20.002 CNY/500 g, respectively. Those in the advanced-age group are also willing to pay a premium of 6.801 CNY/500 g for the entertainment environment attribute. As we expected, highly educated and high-income consumers are the focus of animal welfare marketing.

## 4. Discussions and Policy Implications

### 4.1. Discussions

This study empirically analyzes consumer preferences for pigs’ welfare farming attributes in terms of feed nutrition, living environment, health care, and activity space. In general, consumers have a significant preference for these attributes and are willing to pay a premium of 2.359–10.477 CNY/500 g (5.27–23.39%). Among them, the premium for the attribute of 100% more space and outdoor access is the highest, reaching 10.477 CNY/500 g (23.39%). These results are generally consistent with other domestic studies. For example, Wang and Wu (2013) [44] examined the consumption preferences of urban residents in Changchun, Beijing, Hangzhou, Hohhot, and Chengdu, China, and found that consumers were willing to pay 2.814 CNY/500 g (11.73%) more for animal welfare pork.

However, the premium paid by Chinese consumers may be lower than that of Western developed countries. Liljenstolpe (2008) showed in a study with a choice experiment that Swedish consumers were willing to pay a 32% premium for outdoor-raised pork [45]. Denver et al. (2017) classified pig welfare as standard, medium, and high based on rearing space, i.e., rearing space stipulated by current legislation (at least 0.65 m^2^ per fattening pig), 30% more space (at least 0.85 m^2^), and 100% more space (at least 1.3 m^2^), respectively [27]. Their study showed that Danish consumers were willing to pay a 17–75% premium for medium-level animal welfare pork over standard animal welfare pork while willing to pay a 14% premium for high-level over medium-level animal welfare pork.

Although there is a gap between consumers’ recognition of animal welfare and their actual purchasing behavior, providing information on animal welfare certification may become an important strategy to meet the differentiated needs of Chinese consumers. Currently, the packaging of fresh meat products in supermarkets, farmers’ markets, and the three online fresh food platforms—JD Fresh, Suning Commerce and Fresh Hema—mainly involves information about animal species, parts of the meat, origin, and brand. There is lack of information regarding the farming methods with which the animals are raised. Consumers have to buy meat products based on experience, i.e., relying on personal observations of meat color, texture, etc. Our study found that consumers were willing to pay a significant premium for animal welfare pork. Policymakers and production suppliers may be able to further increase consumer confidence and product premiums by releasing information on the details of good production processes for meat products.

How can consumers be provided with better information for making these decisions? Information regarding animal welfare pork is well trusted by the populace [11]. Information regarding animal welfare attributes can be a useful tool to indicate the high-quality nature of the product if it is available to consumers through markings on the packaging [46]. Many studies have shown that product labeling is an effective tool to ensure that food products meet the individual needs of consumers, e.g., as in Gracia et al. (2011) and Kehlbacher et al. (2012) [47,48]. Therefore, product labeling is increasingly becoming an important regulatory strategy in the EU, especially as it relates to food safety issues [49]. Mandatory labeling, on the other hand, may lead to a negative selection of products with low animal husbandry standards, thus reducing consumers’ choices. However, mandatory or enhanced legislation can improve consumer welfare by increasing the private value of animal welfare meat products [50].

An additional question is how to increase the market supply of animal welfare products. Many studies on willingness to pay for animal welfare have suggested potential strategies for improving the market supply of animal welfare products. However, there are still relatively few animal welfare products in the market, with the exception of a small number of countries such as Switzerland, the UK, and the Netherlands. This suggests that stakeholders in the food supply chain are very important for the improvement of animal welfare.

As pointed out by Thorslund et al. (2017), many steps have to be taken to improve farm animal welfare [51]. First, agribusinesses must be willing and able to produce to higher welfare standards. Second, there must be economic incentives to enable firms to gain, or at least not lose, revenue through animal welfare farming. Third, other entities such as slaughterhouses and meat processors must be willing to sell special products with animal welfare labels. Furthermore, retailers must be willing to market and sell the products. Lastly, consumers must be willing to buy the product at a premium price. In any case, the supply of animal welfare products requires changes in the governance structure between agricultural and production organizations in the food value chain. For example, the issue of animal welfare has to permeate the entire value chain when it cannot be solved at the end-handling stage, which in turn necessitates changes in the relationships between value chain members. In addition, new forms of contractual arrangements between farms and processors need to be established when animal welfare farming methods have been differentiated at the farm (rearing) stage.

Farmers or animal breeding enterprises are the most important stakeholder group for the improvement of animal welfare. Another reason for the low market share of farm animal welfare products may be that producers have doubts about animal welfare. Although many of them have positive attitudes toward farm animal welfare, previous studies have shown that only a minority of farmers recognize the need to improve the level of animal welfare in livestock production systems [25,52]. Practically, production system adjustments may entail high economic risks for producers, i.e., the cost of investment in improving animal welfare may not be matched by a return and selling the product at a higher price may itself be a big problem [53,54,55]. It has also been shown that farmers’ attitudes are closely related to their behavior of improving animal welfare [56,57]. Future investigation of farmers’ attitudes toward animal welfare farming is essential to understand their actual willingness to participate in improving animal welfare and to increase the market supply of animal welfare products in China.

### 4.2. Policy Implications

China is the world’s largest producer and consumer of meat. The present study potentially contributes not only to promoting the high-quality development of animal husbandry, but also to promoting the transformation and upgrading of consumption structure. The following suggestions can be made for meat production suppliers.

The first suggestion is to adjust the mix of improved animal welfare production conditions. In developing product differentiation policies to address the issue of public claims for animal welfare, animal welfare preference must be correlated with production costs. For producers, the benefit–cost ratio of improving breeding conditions may vary greatly. Producers may gradually improve the animal production environment and adjust animal feeding methods according to the differences in consumers’ concerns about animal welfare and their preferences for different welfare breeding conditions. In addition, they may choose to prioritize animal welfare improvements by considering the farm’s own advantageous conditions.

The second suggestion is to establish innovative contractual arrangements between industry chain stakeholder groups. Farmers or enterprises are often bound by contracts at the downstream production stage. It is necessary to involve the slaughter and processing industries in the development of animal welfare standards so that farmers or enterprises have the opportunity to operate under higher animal welfare standards. Additionally, compliance with higher animal welfare standards is a long-term capital investment, and producers may be able to leverage the financial guarantees provided by the downstream industry both to gain the opportunity to produce a high-quality product and to escape the financial pressures of improving animal welfare on their farms by increasing the profitability of their animal products.

The third suggestion is to develop marketing strategies to differentiate animal welfare products. Achieving better economic outcomes has always been the main motivation for farmers to improve animal welfare. Manufacturers need to provide sufficient product information to guide consumers to take responsibility and purchase animal welfare products rather than just treating animal welfare as a problem that needs to be addressed through regulation. In addition, the consumers who buy animal welfare products are not homogeneous. It is important to consider the heterogeneity in consumer preferences during the market launch process, to segment the product market, and to improve the valuation of and demand for animal products with higher-than-average welfare production conditions from different consumer groups so as to obtain the best cost-benefit ratio.

## 5. Conclusions

The issue of animal welfare is still not commonly recognized and there are currently no farm animal welfare-certified products in China. However, the Chinese government has recently launched programs to improve animal welfare. For example, China approved the establishment of the Animal Welfare International Cooperation Committee of the China Association for the Promotion of International Cooperation in Agriculture in 2013. Since then, more regulations and policies have been introduced, such as Farm Animal Welfare Requirements for Pigs (2014), Meat Sheep (2015), Chicken (2017), Laying Hen (2017), Cashmere Goat (2020), and Cows (2021). The process of promoting animal welfare development in China is gradually accelerating. Soon, a widening range of animal-friendly products will be available to meet the consumer demand in China.

Improving animal welfare may lead to increased production costs. If consumers have a willingness to pay for farm animal welfare, it will help strengthen the determination and confidence of producers to improve animal welfare. In this regard, this paper examined the consumer preferences for animal welfare pork using data from a choice experiment with 1274 pork consumers in Guangdong province. The results showed that consumers had a significant payment premium of 2.359–10.477 CNY/500g (5.27–23.39%) for pork with different animal welfare characteristics. Products with the “100% more space and outdoor access” animal welfare attribute are the most valued, followed by “optimal care”, “fermented feed”, “recreational environment”, and “100% more space” attributes. The study found no significant gender differences in consumer preferences, but age, education, and income all had varying degrees of influence on animal welfare pork consumption preference. The empirical findings are useful to both industry practitioners and decision-makers in promoting the transition to more sustainable animal welfare farming practices in society.

Our study has some limitations, which should be addressed by future research. Our survey was conducted at the beginning of the global COVID-19 outbreak in 2020, when quarantine and isolation rules were implemented in China. Further research is needed to determine whether people’s consumption habits for products with animal welfare attributes could change before and after the pandemic. Additionally, despite the fact that the scope of our study is limited to China, these results may produce useful pieces of information that might help developing countries creating policies to improve animal welfare and enhance their competitiveness in international trade of livestock products.

## Figures and Tables

**Table 1:** Farm animal welfare attributes and their levels in the choice experiment.

Attribute	Level	Description
Feed nutrition	Common feed	The current national standards for nutrient content are met.
	Fermented feed	The current national standards for nutrient content are met. In addition, the feed is enriched with probiotics (which help intestinal digestion and reduce food residue).
Living environment	Standard environment	The air, ventilation, and other environmental parameters of the pig house are in line with the national standards.
	Recreational environment	The air, ventilation, and other environmental parameters of the pig house are in line with the national standards. In addition, toys, music, and other recreational facilities are provided.
Health care	Basic care	Basic, necessary epidemic diagnosis and treatment are provided.
	Optimal care	Measures such as frequent disinfection and disease monitoring are taken. Veterinarians provide a daily inspection and a timely diagnosis and treatment of sick or injured pigs. Pain-free surgery is given to avoid pain unrelated to the disease.
Activity space	Standard space	Indoor space in accordance with the national standard is at least 0.8–1.2 m^2^ of bedding area per pig.
	100% more space	According to the national standard, 100% more indoor space takes up at least 1.6–2.4 m^2^ of bedding area per pig.
	100% more space and outdoor access	100% more indoor space takes up at least 1.6–2.4 m^2^ of bedding area per pig. In addition, access to outdoor pasture is provided.
Price	44.8, 54.8, 64.8, 74.8	These are the prices at which the respondents usually bought fresh lean pork in supermarkets or wet markets (unit: CNY/500 g).

**Table 2:** Some socio-demographic characteristics of respondents (n = 1274).

Socio-Demographics		Socio-Demographics	
Gender (%)		Monthly household income (%)	
Male	37.52	<6000 CNY	17.03
Female	62.48	6000–12,000 CNY	33.28
Age (in years) Mean (s.e.)	32.25 (9.998)	12,000–18,000 CNY	20.88
Education level (%)		18,000–24,000 CNY	13.34
Primary school and below	0.55	24,000–30,000 CNY	8.01
Junior high school	5.18	>30,000	7.46
High school/technical secondary school)	10.08	Number of dining members (%)	
College/higher vocational	16.64	≤2	17.04
Undergraduate	54.00	3	24.88
Postgraduate and above	13.58 ^1^	4	26.14
Eat with children under 18 years old (%)	49.69	5	21.11
Eat with the elderly above 60 years old (%)	39.87	≥6	10.83

^1^ Note: Percentages may total >100% because of rounding.

**Table 3:** Random parameter logit results of consumer preferences for animal welfare pork.

Variables	Mean	Standard Deviation	Willingness to Pay	Willingness to Pay (%)
Fermented feed	0.380 ***	0.372 ***	5.893	13.15
	(0.033)	(0.075)	[4.912, 6.874]	
100% more space	0.152 ***	−0.027	2.359	5.27
	(0.041)	(0.102)	[1.100, 3.618]	
Increase 100% space and outdoor access	0.676 ***	−0.229	10.477	23.39
	(0.045)	(0.154)	[9.114, 11.839]	
Optimal care	0.431 ***	0.434 ***	6.689	14.93
	(0.034)	(0.067)	[5.640, 7.737]	
Recreational environment	0.165 ***	0.754 ***	2.560	5.71
	(0.036)	(0.056)	[1.455, 3.666]	
Would not buy	−6.770 ***	2.849 ***		
	(0.234)	(0.169)		
Price	−0.064 ***			
	(0.002)			
Number of observations	22,932			
LR chi2	848.02			
Log likelihood	−5684.849			
AIC	11,395.7			

*** indicates statistical significance at the 1% level. The numbers in parentheses are standard errors, and the numbers in brackets are 95% confidence intervals. Willingness to pay (%) calculates the proportion of payment premiums obtained through the RPL model, i.e., the ratio of WTP to the base price (44.8 CNY/500 g) in the choice set, so as to facilitate comparison of the proportion of price premiums between different products in the existing literature.

**Table 4:** Random parameter logit results with socio-demographics interaction terms.

Variables	With Gender Interaction	With Age Interaction		With Education Interaction	With Income Interaction
Price	−0.064 ***	−0.065 ***		−0.065 ***	−0.065 ***
	(0.002)	(0.002)		(0.002)	(0.002)
Fermented feed	0.393 ***	0.347 ***		0.246 ***	0.252 ***
	(0.052)	(0.055)		(0.055)	(0.044)
100% more space	0.142 **	0.146 **		0.106	0.121 **
	(0.067)	(0.071)		(0.070)	(0.057)
100% more space and outdoor access	0.603 ***	0.572 ***		0.602 ***	0.618 ***
	(0.070)	(0.074)		(0.074)	(0.061)
Optimal care	0.440 ***	0.512 ***		0.344 ***	0.386 ***
	(0.053)	(0.057)		(0.056)	(0.046)
Entertainment environment	0.166 ***	0.240 ***		0.174 ***	0.114 **
	(0.059)	(0.063)		(0.062)	(0.050)
No purchase	−6.768 ***	−6.773 ***		−6.776 ***	−6.745 ***
	(0.234)	(0.234)		(0.236)	(0.232)
Interaction items between attributes and socio-demographics	Female	Middle Age (26–35)	Advanced Age (≥36)	High Education (≥16)	High Income (≥12,000)
Fermented feed ×	−0.021	0.088	0.002	0.196 ***	0.253 ***
	(0.065)	(0.075)	(0.079)	(0.067)	(0.063)
100% more space ×	0.016	−0.026	0.044	0.063	0.060
	(0.083)	(0.098)	(0.100)	(0.086)	(0.081)
100% more space and outdoor access ×	0.117	0.105	0.202 *	0.109	0.116
	(0.086)	(0.100)	(0.105)	(0.088)	(0.083)
Optimal care ×	−0.015	−0.134 *	−0.103	0.126 *	0.086
	(0.066)	(0.077)	(0.080)	(0.068)	(0.064)
Recreational environment ×	−0.001	−0.095	−0.128	−0.017	0.102
	(0.074)	(0.086)	(0.090)	(0.076)	(0.072)
Number of observations	22,932	22,932		22,932	22,932
Wald chi2	−5683.6497	−5678.736		−5678.379	−5673.9201
Log likelihood	847.75	847.31		846.94	831.47
AIC	11,403.3	11,403.47		11,392.76	11,383.84

Note: ***, **, and * indicate statistical significance at the 1%, 5%, and 10% levels, respectively. The numbers in parentheses are standard errors. The standard deviation of random parameters for each attribute variable is not reported in this paper due to space limitations.

**Table 5:** Random parameter logit results of consumer preferences by six groups.

Variables	Lower Education (<16 Years), Lower Income (<12,000 CNY)			Higher Education (≥16 Years), Higher Income (≥12,000 CNY)		
	Low Age (≥25)	Middle Age (26–35)	Advanced Age (≥36)	Low Age (≥25)	Middle Age (26–35)	Advanced Age (≥36)
Price	−0.178 ***	−0.052 ***	−0.035 ***	−0.139 ***	−0.071 ***	−0.059 ***
	(0.037)	(0.013)	(0.007)	(0.016)	(0.007)	(0.011)
Fermented feed	0.518	0.346 *	0.095	0.865 ***	0.512 ***	0.692 ***
	(0.520)	(0.194)	(0.116)	(0.212)	(0.116)	(0.163)
100% more space	−0.571	−0.058	0.239 *	−0.008	0.154	0.440 **
	(0.426)	(0.344)	(0.136)	(0.208)	(0.128)	(0.180)
100% more space and outdoor access	0.366	0.377	0.669 ***	0.773 ***	0.836 ***	1.175 ***
	(0.451)	(0.302)	(0.151)	(0.231)	(0.148)	(0.239)
Optimal care	0.026	0.710 ***	0.359 ***	0.900 ***	0.513 ***	0.685 ***
	(0.374)	(0.235)	(0.108)	(0.209)	(0.107)	(0.156)
Recreational environment	0.198	0.123	−0.052	0.263	0.096	0.399 **
	(0.491)	(0.220)	(0.123)	(0.189)	(0.109)	(0.189)
Would not buy	−12.425 ***	−5.041 ***	−5.732 ***	−11.567 ***	−8.231 ***	−7.541 ***
	(2.499)	(1.105)	(0.885)	(1.337)	(1.005)	(1.533)
Number of observations	522	738	1836	1638	2664	1332
Wald chi2	44.53	51.88	94.72	68.89	91.25	59.63
Log likelihood	−116.292	−196.791	−487.112	−335.2545	−616.9963	−299.408
AIC	258.584	419.582	1000.223	696.509	1259.993	624.8168

***, **, and * indicate statistical significance at the 1%, 5%, and 10% levels, respectively. The numbers in parentheses are standard errors. The standard deviation of random parameters for each attribute variable is not reported in this paper due to space limitations.

**Table 6:** Estimates of consumers’ willingness to pay by six groups.

	Fermented Feed	100% More Space	100% More Space and Outdoor Access	Optimal Care	Recreational Environment
Lower income, lower education					
Income = 6000 CNY, education = 9 years					
Age = 22.379	2.906	−3.202	2.053	0.144	1.112
	[−2.419, 8.230]	[−7.772, 1.369]	[−2.896, 7.003]	[−3.971, 4.259]	[−4.205, 6.430]
Age = 32.250	6.677 *	−1.122	7.276	13.715 ***	2.375
	[−0.686, 14.040]	[−14.066, 11.823]	[−4.772, 19,325]	[4.447, 22.984]	[−6.085, 10.836]
Age = 45.216	2.743	6.909 *	19.335 ***	10.391 ***	−1.503
	[−3.832, 9.318]	[−0.975, 14.792]	[9.510, 29.161]	[3.430, 17.351]	[−8.438, 5.433]
Higher income, higher education					
Income = 24,000 CNY, education = 16 years					
Age = 22.379	6.204 ***	−0.056	5.541 ***	6.454 ***	1.889
	[3.643, 8.764]	[−2.971, 2.860]	[2.498, 8.584]	[3.636, 9.272]	[−0.715, 4.493]
Age = 32.250	7.730 ***	2.175	11.792 ***	7.240 ***	1.35
	[4.052, 10.408]	[−1.387, 5.737]	[7.815, 15.769]	[4.355, 10.125]	[−1.668, 4.367]
Age = 45.216	11.780 ***	7.499 **	20.002 ***	11.671 ***	6.801 **
	[6.465, 17.095]	[1.261, 13.737]	[11.671, 28.333]	[5.565, 17.777]	[0.553, 13.049]

***, **, and * indicate statistical significance at the 1%, 5%, and 10% levels, respectively. The numbers in brackets are 95% confidence intervals.

## Data Availability

All study data used for analysis are available upon request.
